# Immunoexpression of Survivin and P53 in the Histological Subtypes of Medulloblastoma: A Cross-Sectional Observational Study

**DOI:** 10.7759/cureus.65627

**Published:** 2024-07-29

**Authors:** Guralarasan G, Shreekant Bharti, Vikas C Jha, Jitendra S Nigam, Abhirami Ganesh R, Punam Bhadani

**Affiliations:** 1 Pathology and Lab Medicine, All India Institute of Medical Sciences, Patna, Patna, IND; 2 Neurosurgery, All India Institute of Medical Sciences, Patna, Patna, IND; 3 Pathology and Lab Medicine, All India Institute of Medical Sciences, Bibinagar, Bibinagar, IND

**Keywords:** pediatric brain tumor, histopathology and immunohistochemistry, p53 expression, survivin protein, pediatric medulloblastoma

## Abstract

Medulloblastoma (MB) is a common malignant intracranial neoplasms in children. The treatment and prognosis of this tumor depends on histology and molecular subtypes. Survivin, implicated in various malignancies, may hold prognostic significance. We investigated survivin and p53 immunoreactivity in different histological subtypes in 20 MB cases from January 2018 to June 2021. Immunohistochemistry revealed survivin expression in 75% (15/20) of cases, with cytoplasmic (10 cases), nuclear (four cases), or combined expression (one case). p53 nuclear expression was present in 35% (7/20) of cases. Classical variant MB exhibited predominant p53 and cytoplasmic survivin expression. Given the association of survivin and p53 expression with poor prognosis, especially in the prevalent classical variant, targeted therapies may hold promise for MB treatment advancement.

## Introduction

Medulloblastoma (MB) is a primary malignant tumour of the central nervous system accounting for 18-20% of all childhood brain tumours. It arises almost exclusively from the cerebellum and accounts for around 1/4th of all intracranial neoplasms and almost half of posterior fossa tumours [1]. It is the most common malignant embryonal neoplasm in the paediatric age group. It is more commonly seen in children younger than 10 years of age but can also occur in adults, with a lesser incidence [2]. According to the WHO Classification of CNS Tumours 2021, MB is categorised as Grade 4 due to its aggressive behaviour. If left untreated, it is a rapidly fatal disease with a five-year overall survival rate of nearly 75% [3]. The treatment and prognosis of MB depend on the histology and molecular subtypes. Molecular classification is not always available or feasible in most laboratories. Moreover, the same histological types have varying prognoses in different individuals [4].

Survivin is a protein that belongs to the inhibitors of apoptosis (IAP) family encoded by the BIRC5 gene located on chromosome 17 [5]. P53 is a nuclear phosphoprotein that regulates DNA replication, cell proliferation, and apoptosis. Even though the role of p53 in the initiation, progression, and maintenance of MB is not understood [6], its immunoreactivity is related to poor prognosis [7]. Hence, immunohistochemical analysis of survivin and p53 protein expression can be a useful tool for the prognostic sub-stratification of patients with MBs.

To date, very few studies have been conducted for the evaluation of both survivin and p53 in MB in Indian populations. In the current work, our objective was to know the level of apoptotic inhibition by assessing the immunoreactivity of survivin and p53 in different histological subtypes of MB.

## Materials and methods

This study was done in the Department of Pathology/Laboratory Medicine during the year 2019 till 2021. The expected sample size of 35 was not met because our hospital was declared a dedicated COVID-19 care centre during the COVID-19 pandemic. A common waiver for not meeting the exact sample size due to the pandemic situation was granted by the Office of the Dean to all the approved research projects in this period (letter no. 476/AIIMS/PAT/Dean/2021). Twenty histologically proven MB cases were examined in this cross-sectional study from January 2018 to June 2021. 

Histopathological analysis

**Figure 1:**
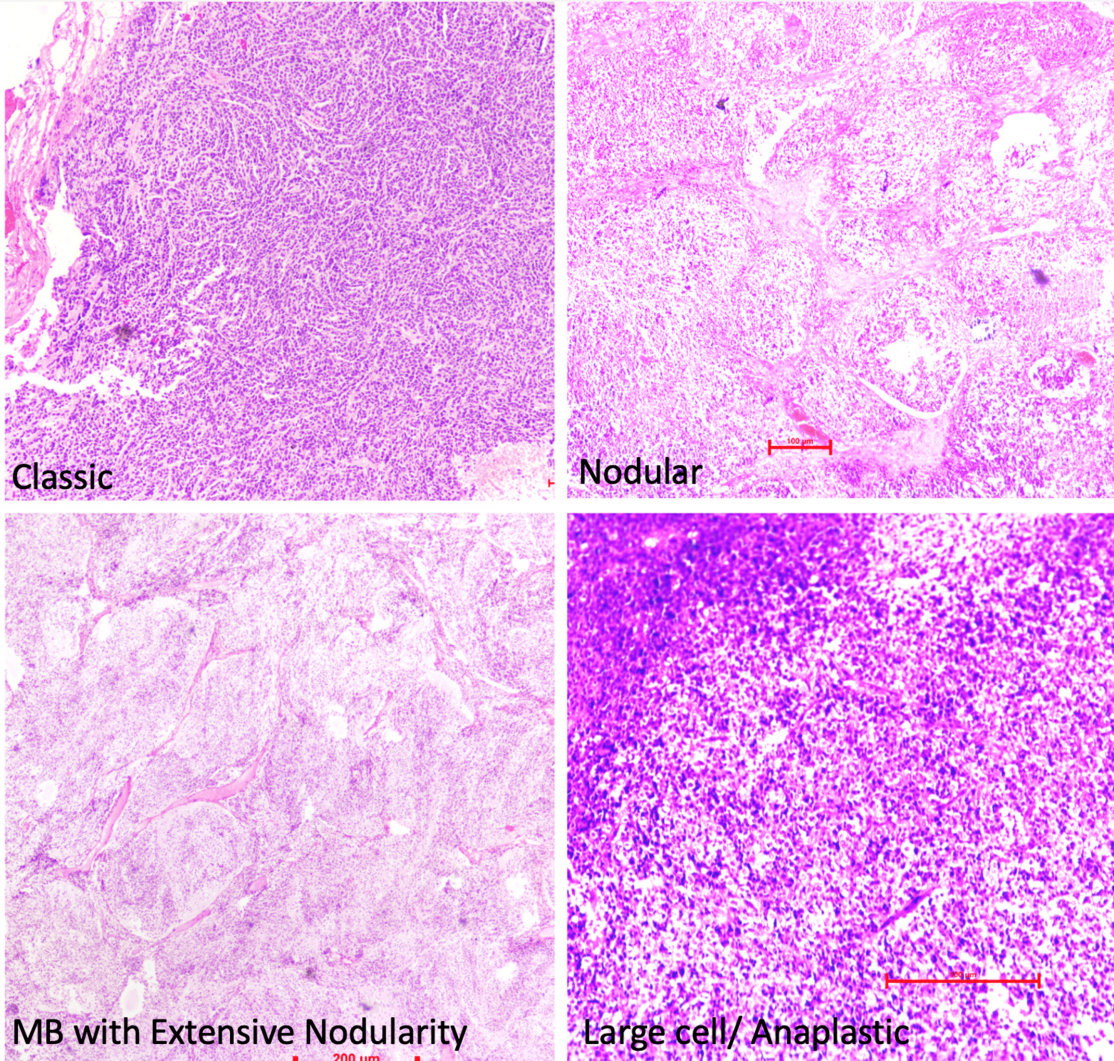
Histopathological subtypes of medulloblastoma (hematoxylin and eosin stain, 200x magnification)

Formalin-fixed paraffin-embedded blocks were retrieved. Hematoxylin and eosin (H&E) and reticulin staining were performed. Slides were re-evaluated and the histopathological diagnosis of MB was made in the light of morphological features, age, site of lesion, and radiological features. This was further supported by immunoreactivity for synaptophysin and chromogranin. Histological subclassification of MBs was made according to the WHO Classification of CNS Tumours into classical, desmoplastic/nodular, MB with extensive nodularity, and anaplastic/large cell types. Representative images of the four histopathological subtypes are shown in Figure 1.

Immunohistochemistry (IHC)

**Figure 2:**
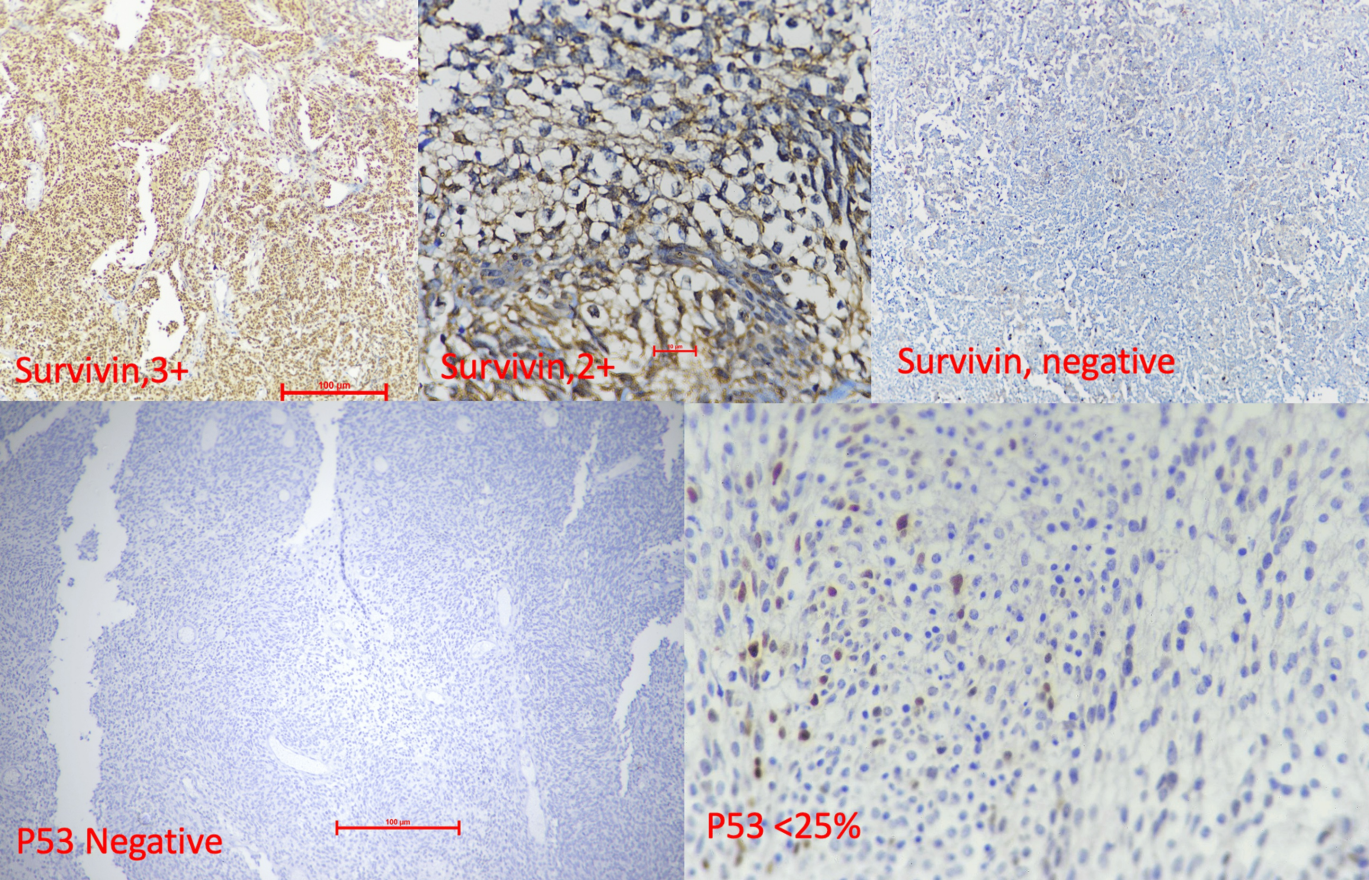
Expression of survivin with different intensities and localizations (top panel). P53 immunostaining (bottom panel) The medulloblastoma cases showed varying intensities and localization of survivin as demonstrated. Strong nuclear positivity of survivin (3+, top left) and moderate cytoplasmic positivity (2+, top right). P53 shows a negative (lower-left panel) and less than 25% nuclear stain (lower-right panel).

IHC staining was done on the representative tumour sections for synaptophysin, survivin, and p53 on an in-house automated IHC stainer (Ventana Benchmark GX, Roche Diagnostics, Switzerland). Representative paraffin blocks were cooled or placed in a refrigerator at 4°C for 20-30 minutes. Then, a 4-5-μm-thick section was cut and transferred to a poly-L-lysine-coated slide. The slide was labelled and left at room temperature for 12 hours. Then, the slide was placed on a hot plate at 70°C for 15-20 minutes. Primary antibodies against survivin D-8 (Mouse Monoclonal SC-17779 HRP Santa Cruz, USA; dilution 1:200), anti-p53 (ready to use, Clone Bp53-11; Mouse Monoclonal, Ventana, USA), and anti-synaptophysin (SP11, RTU Rabbit Monoclonal; Ventana, USA) were applied (Figure 2).

IHC scoring

IHC-stained slides were independently evaluated and scored by three experienced pathologists. Two pathologists evaluated and diagnosed the cases with independent IHC scoring. Any discrepant scoring was resolved by the scoring given by a third senior pathologist and values within the same score category by at least two pathologists were accepted. The IHC results were assessed and graded on a four-tier scale. The percentage and intensity of the proportion of survivin and p53-positive cells were estimated. Evaluation of the studied cases was semi-quantitative depending on nuclear/cytoplasmic staining and was scored according to the percentage and intensity of positive nuclei to the total cells counted/10 hpf [8,9]. Percentage categories were 0, 1-25%, 25-50%, and >50%. The intensity was graded as no staining (0), weak (1), moderate (2), and strong (3). Survivin and p53 scoring (percentage of cells + intensity) ranged from 1 to 6.

## Results

All cases were reviewed thoroughly to determine the clinicopathological characteristics of the MB, namely, age, sex, presenting complaints, histological types, and grade of the tumour. Haematoxylin and eosin and reticulin staining were done to assess the cellular morphology and histological pattern for further histological subtyping of the MB into the four histological types mentioned above. Synaptophysin IHC was done to differentiate it from other posterior fossa tumours and confirm MB. The median age of diagnosis of MB was eight years. Five cases (25%) were less than five years, eight cases (40%) were between five and 10 years, four cases (20%) were between 10 and 15 years, and three cases were more than 15 years. Out of 20 cases, 14 cases (70%) were males and six cases (30%) were females. The predominant subtype was the classical variant (n = 12, 60%), followed by three cases (15%) each of desmoplastic/nodular and MB with extensive nodularity subtypes. Two cases (10%) were of large cell/anaplastic subtype (Table 1).

**Table 1:** Distribution of various histological subtypes among different age groups (N = 20)

Age in years	Classic (n/%)	Desmoplastic/nodular (n/%)	MB with extensive nodularity (n/%)	Large cell/anaplastic (n/%)
0-5	2 (40%)	1 (20%)	2 (40%)	0
5-10	5 (62.5%)	1 (12.5%)	1 (12.5%)	1 (12.5%)
10-15	2 (50%)	1 (25%)	0	1 (25%)
15-20	2 (100%)	0	0	0
25-30	1 (100%)	0	0	0
Total	12 (60%)	3 (15%)	3 (15%)	2 (10%)

Expression patterns of survivin

Survivin showed both nuclear and cytoplasmic expressions. Out of 20 cases, 15 cases showed cytoplasmic or nuclear immunopositivity for survivin. In those 15 cases, 10 cases (66.66%) belonged to the classic subtype, two cases (13.33%) belonged to the desmoplastic/nodular subgroup, two cases (13.33%) belonged to MB with extensive nodularity, and one case (6.66%) belonged to the large cell/anaplastic variant. Out of 20 cases, 10 cases (66.66%) showed a cytoplasmic expression, four cases (26.66%) showed a nuclear expression, and one case (6.66%) showed both nuclear and cytoplasmic expressions (Table 2).

**Table 2:** Localization of survivin in the histological subtypes of MB (N = 20)

Survivin localization	Classic (n/%)	Desmoplastic/nodular (n/%)	MB with extensive nodularity (n/%)	Large cell/anaplastic (n/%)	Total (n/%)
Nuclear	4 (33.3%)	0	0	0	4 (20%)
Cytoplasmic	5 (41.7%)	2 (66.7%)	2 (66.7%)	1 (50%)	10 (50%)
Nuclear and cytoplasmic	1 (8.3%)	0	0	0	1 (5%)
Negative	2 (16.7%)	1 (33.3%)	1 (33.3%)	1 (50%)	5 (25%)

Nuclear localization of survivin in the histological subtypes of MB

Of these five nuclear-positive MBs, two (40%) cases showed a positive tumour cell percentage of more than 50, one case showed a positive tumour cell percentage between 25% and 50%, and two cases (40%) between 1% and 25%. Out of five cases, two cases (40%) showed strong intensity, two cases (40%) showed moderate intensity, and one case (20%) showed weak positivity. The score was given by adding positive tumour percentage category and intensity, assigned 0-6 for nuclear expression, and two cases had the highest score of 6. The remaining three cases each had scores of 2, 3, and 4.

Cytoplasmic localization of survivin in the histological subtypes of MB

Eleven MB cases showed a cytoplasmic expression. Depending on the percentage of positive tumour cells, they were grouped into three categories, 1-25%, 25-50%, and more than 50%. Out of these 11 cases, seven cases (63.60%) showed more than 50% of positive tumour cells, two cases (18.18%) showed positive cells between 25% and 50%, and two cases (18.18%) showed between 1% and 25%. The cytoplasmic expression was seen in 11 cases. Of those, six cases were classic, two cases were desmoplastic/nodular, another two cases were MB with extensive nodularity, and one case was a large cell/anaplastic variant. Four cases showed positive cells in more than 50%, one case in 25-50%, and one case with <25%. Among two desmoplastic/nodular subtypes, one case had <25% and one case showed >50% of positive tumour cells. MB with extensive nodularity showed cytoplasmic positivity in two cases, which both had a percentage of positive tumour cells >50. One case of a large cell/anaplastic variant showed a percentage category between 25 and 50. Six cases (55%) showed strong intensity and five cases (45%) showed moderate intensity. Four cases (36.36%) had a score of 6, and four cases (36.36%) had a score of 5. Two cases (18.18%) had a score of 4, and one case (9.09%) had a score of 3.

Expression of p53

Regarding p53 expression, 13 cases (65%) did not show any expression, and the remaining seven cases (35%) showed positive tumour cell percentages ranging from 1 to 25. Two were of the classic subtype, two were desmoplastic/nodular, two were MB with extensive nodularity, and one was was of the large cell/anaplastic subtype. Out of seven cases with p53 expression, five cases (25%) had a score of 3 and two cases (10%) had a score of 2. Classic and desmoplastic/nodular subtypes had a score of 3. Two cases of MB had extensive nodularity, among which one was a score of 3 and the other was a score of 2. One case of large cell/anaplastic subtype showed a score of 2.

Statistical analysis

A chi-square test of independence was conducted between histological subtypes of MB with a p53 score and survivin cytoplasmic score separately. The majority of cells showed expected frequencies of less than 5. Fisher's exact test values were p = 0.076 (for p53) and p = 0.665 (for survivin), which showed that there was no significant association between the p53 score and histological subtype of MB, as well as the survivin cytoplasmic score and histological subtype of MB.

## Discussion

MB is the most common CNS embryonal tumour in children, accounting for 30-40% of all brain tumours in children [10]. To provide an 'integrated diagnosis', the WHO Classification of CNS Tumours 2021 update recommends layering diagnoses with histological classification, WHO grading, and molecular/genetic subgroups. Although histological classification remains unchanged, molecular classification provides more clinical and prognostic information than histopathological examination alone [3]. As already discussed, the treatment and prognosis of MB depend on the histology and molecular subtypes.

Survivin is a protein of the IAP family involved in the inhibition of apoptosis and regulation of the cell cycle. These functional attributes make survivin a unique protein exhibiting divergent functions, i.e., regulating cell proliferation and cell death. The anti-apoptotic effect of survivin and its influence on cell division is a result of a complex intermolecular interaction within the nuclear and cytosolic compartments. Its anti-apoptotic effect is produced by conferring an increased survival threshold to cells, effect on multiple molecular pathways of mitochondria homeostasis, and translational and post-translational modification dynamics. The cell cycle regulation effects are broadly related to its role as a part of the chromosomal passenger complex affecting other molecules of this complex, mainly aurora kinase B (AURKB), inner centromere protein antigens (INCENP), and borealin. Survivin also interacts with mitotic spindle formation, microtubule assembly, and cell cycle checkpoint proteins affecting cell division [11].

Survivin expression in WNT MBs is generally lower compared to other subgroups. This lower expression correlates with the favourable prognosis and less aggressive nature of WNT tumours. The SHH and group 3 molecular subtypes show high survivin expression. Expression of survivin is not only correlated with a decreased rate of cell death but also resistance to chemotherapy, aggressiveness of the tumour, and thus a bad prognosis [4,11].

It is a well-established fact that being the guardian of the genome, mutant p53 is associated with poor prognosis not only in MB but also in other cancers [12].

In this study, we analysed survivin and p53 expression and its association across various histological subtypes of MB. The mean patient age at the time of diagnosis was around eight years with peak incidence at five to 10 years and a male preponderance (M:F ratio is 2.3:1). This was similar to the study done by Roberts et al. [13] and Haberler et al. [14]. On histopathological subtyping, as defined by the WHO Classification of Tumours of CNS 2021, the predominant histological subgroup was of the classical variant, followed by the desmoplastic/nodular variant and MB with extensive nodularity and lastly the anaplastic/large cell variant, similar to Kaur et al. [15], Ellison et al. [16], and Kool et al. [17].

Expression of survivin in MB

In our study, survivin expression (nuclear and/or cytoplasmic) was seen in 75% of cases (15/20) while 25% (5/20) cases were negative. It was comparable to a similar study of survivin expression conducted by Sasaki et al. [8]. Five out of 20 cases showed nuclear expression of survivin, whereas 75% (16/20) cases had no nuclear expression. All five cases that showed nuclear expression were exclusive of the classic variant of MB similar to the study done by Aziz et al. [9] and Li et al. [18]. Pizem et al. [19] in their study found nuclear positivity for survivin in all the cases.

In our study, 55% (11/20) of the cases showed the cytoplasmic expression of survivin. Studies done by Li et al. [18] and Faccion et al. [20] reported cytoplasmic positivity at 25% and 24.4%, respectively, which was less than our study results.

Expression of p53 in MB

Out of the 20 cases analysed, p53 expression was seen in seven cases (35%), while Ray et al. [21] and Tabori et al. [22] showed 14.28% and 12% cases with p53 expression, respectively. In studies conducted by Tabori et al. [22] and Ray et al. [21], 85.7% and 88% of cases were p53-negative, respectively. TP53 mutant cases often have poor overall survival and are resistant to conventional chemo- and radiotherapy.

Among the various histological variants, p53 expression and cytoplasmic expression of survivin in this study were more exclusively seen in the classical variant. This differs from the limited data available on survivin where the nuclear expression was more frequently seen in the anaplastic type. This might be because of the small sample size of our study due to COVID-19 limitations.

## Conclusions

Survivin, being an inhibitor of apoptosis, carries a worse prognosis when overexpressed. The cytoplasmic expression of survivin was more common in our study than the nuclear expression, while p53 was negative in three-fourths of the cases. Cytoplasmic overexpression as compared to the nuclear expression of survivin and mutant p53 is associated with poor prognosis in MB. Targeted therapies are under trial for it. Survivin can serve as a potential prognostic biomarker for oncologists and can help in patient selection for targeted therapeutic trials. More detailed studies are required to explore this horizon.
